# Tau accumulation in degradative organelles is associated to lysosomal stress

**DOI:** 10.1038/s41598-023-44979-7

**Published:** 2023-10-21

**Authors:** Ester Piovesana, Claudia Magrin, Matteo Ciccaldo, Martina Sola, Manolo Bellotto, Maurizio Molinari, Stéphanie Papin, Paolo Paganetti

**Affiliations:** 1 Laboratory for Aging Disorders, Laboratories for Translational Research, Ente Ospedaliero Cantonale, Bellinzona, Switzerland; 2 PhD Program in Neurosciences, Faculty of Biomedical Sciences, Università della Svizzera Italiana, Lugano, Switzerland; 3 Institute for Research in Biomedicine, Faculty of Biomedical Sciences, Università della Svizzera italiana, Bellinzona, Switzerland; 4 GT Gain Therapeutics SA, Lugano, Switzerland; 5 School of Life Sciences, École Polytechnique Fédérale de Lausanne, Lausanne, Switzerland; 6 Neurocentro della Svizzera Italiana, Ente Ospedaliero Cantonale, Lugano, Switzerland

**Keywords:** Molecular neuroscience, Mechanisms of disease

## Abstract

Neurodegenerative disorders are characterized by the brain deposition of insoluble amyloidogenic proteins, such as α-synuclein or Tau, and the concomitant deterioration of cell functions such as the autophagy-lysosomal pathway (ALP). The ALP is involved in the degradation of intracellular macromolecules including protein aggregates. ALP dysfunction due to inherited defects in lysosomal or non-lysosomal proteins causes a group of diseases called lysosomal storage disorders (LSD) because of abnormal accumulation of lysosomal degradation substrates. Supporting the contribution of ALP defects in neurodegenerative diseases, deposition of amyloidogenic proteins occurs in LSD. Moreover, heterozygous mutations of several ALP genes represent risk factors for Parkinson’s disease. The reciprocal contribution of α-synuclein accumulation and lysosomal dysfunction have been extensively studied. However, whether this adverse crosstalk also embraces Tau pathology needs more investigation. Here, we show in human primary fibroblasts that Tau seeds isolated from the brain of Alzheimer’s disease induce Tau accumulation in acidic degradative organelles and lysosomal stress. Furthermore, inhibition of glucocerebrosidase, a lysosomal enzyme mutated in Gaucher’s disease and a main risk for Parkinson’s disease, causes lysosomal dysfunction in primary fibroblasts and contributes to the accumulation of Tau. Considering the presence of Tau lesions in Parkinson’s disease as well as in multiple neurodegenerative disorders including Alzheimer’s disease, our data call for further studies on strategies to alleviate ALP dysfunction as new therapeutic opportunity for neurodegenerative diseases and LSD.

## Introduction

A hallmark of neurodegenerative disorders is the deposition of insoluble protein aggregates composed of amyloidogenic proteins in the brain^1^. The most frequent neurodegenerative disorder is Alzheimer’s disease (AD), characterized by the progressive formation of amyloid plaques composed of amyloid-beta peptides and of neurofibrillar hyperphosphorylated Tau protein aggregates^2,3^. In contrast, α-synuclein deposition within Lewy bodies and neurites characterizes Parkinson’s disease (PD), which also presents Tau lesions^4^. Protein deposits are linked to a decline of cell functions culminating in cell death. However, cellular functions deteriorate at lower pace also during normal aging. An instance is the autophagy-lysosomal pathway (ALP), a cell process dedicated at the degradation of intracellular macromolecules and organelles^5,6^. ALP can also eliminate protein aggregates and so, its dysfunction is proposed to contribute to the neurodegenerative process^7,8^. In support of this is the evidence of increased deposition of amyloidogenic proteins in lysosomal storage disorders (LSD). LSD are caused by inherited defects in lysosomal or non-lysosomal proteins resulting in aberrant buildup of lysosomal substrates and deleterious ALP dysfunction^9^.

The correlation between protein accumulation and lysosomal dysfunction is better documented in PD compared to AD. Some mendelian forms of PD result from mutations in ALP genes e.g., ATP13A2^10^, LRRK2^11^ or VPS35^12^. Also, monoallelic ALP gene mutations represent risk factors for PD but cause specific LSD in homozygote mutation carriers, validating the link between PD and LSD^13,14^. Illustrative is the example of the *GBA1* gene encoding for glucocerebrosidase (GCase), a lysosomal enzyme metabolizing glucosylceramide. Biallelic *GBA1* mutation causes Gaucher’s disease, mendelian disorders affecting several organs and tissues due to cells accumulating fatty substances. In contrast, monoallelic *GBA1* mutations are present in ~ 10% of the patients affected by PD, representing thus the main genetic risk for PD and linking GCase dysfunction to α-synuclein accumulation^15^. However, healthy *GBA1* mutation carriers also show aberrant sphingolipids metabolism and α-synuclein accumulation^16,17^.

Lower GCase activity and accumulation of glucosylceramide in lysosomes can inhibit autophagy^18^ and favor the formation of soluble oligomeric α-synuclein intermediates that can be converted into deposited amyloid fibrils^19^. Aggregation of α-synuclein can impair the trafficking of newly synthesized GCase i.e., reducing the amount of GCase reaching its final destination in lysosomes. In addition, GCase can bind to α-synuclein both in solution and on cell membranes^20^, which affects both GCase activity and the access to GCase substrates^21^. Therefore, the loss of functional GCase creates a noxious circle of glucosylceramide and α-synuclein accumulation that ultimately leads to ALP dysfunction and neurodegeneration^19^. Despite similar aggregation and spreading properties, the relationship between Tau aggregation and ALP deficiency has been less intensively investigated so far. In the current study, we employed AD brain-derived Tau seeds together with a specific GCase inhibitor to describe that ALP impairment contributes to Tau accumulation in degradative organelles of primary human fibroblasts. However, *GBA1* mutations are not associated to AD, possibly suggesting a key role of α-synuclein accumulation in human tauopathies^22,23^.

## Results

### Generation and characterization of human fibroblast lines expressing Tau fused to fluorescent proteins

**Figure 1:**
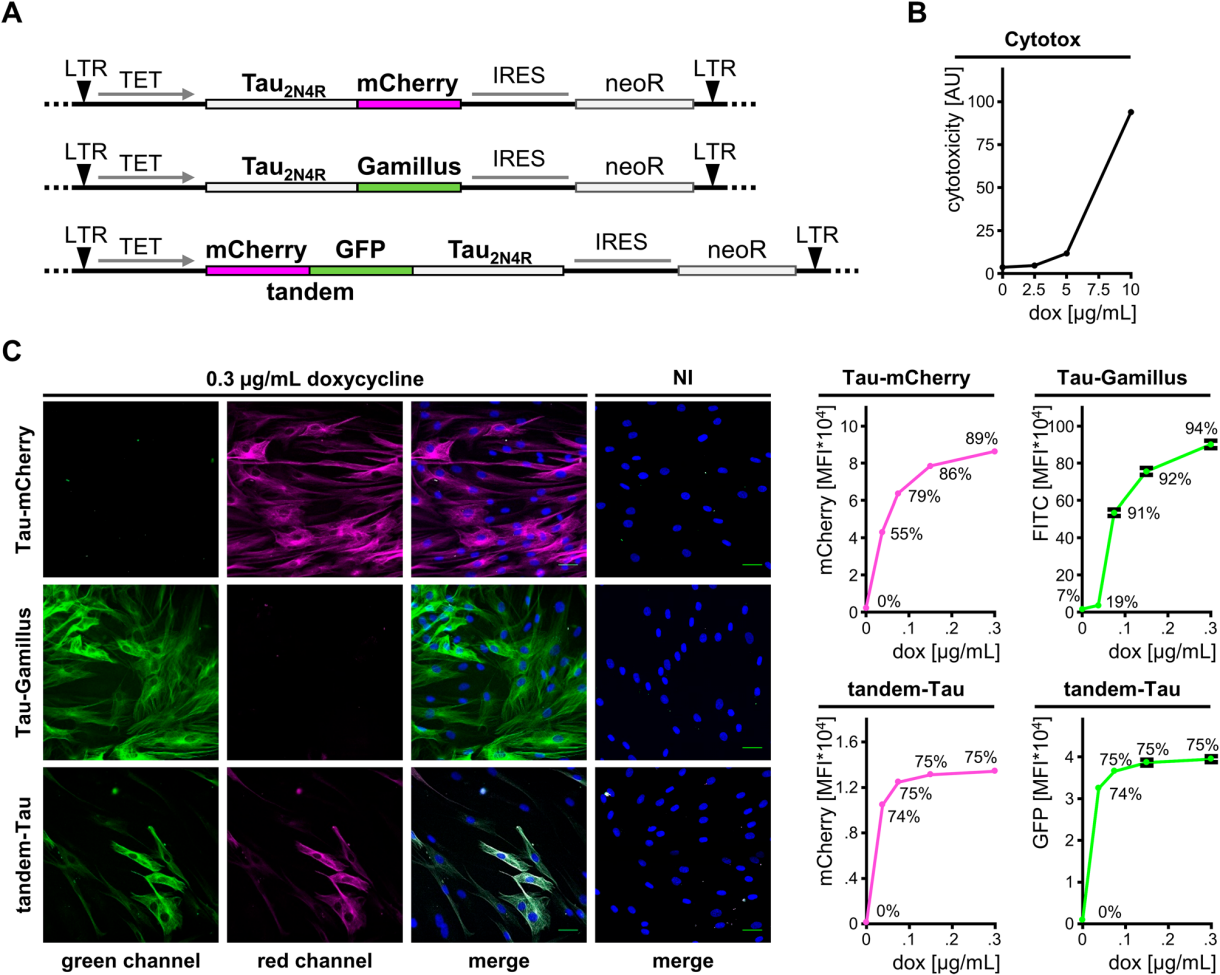
Inducible Tau-expression in primary human fibroblasts. (**A**) Design of the lentiviral constructs driving the inducible expression of Tau-mCherry, Tau-Gamillus and tandem-Tau. (**B**) Parental fibroblasts treated for 2 days with doxycycline were analyzed by cytofluorimetry for cytotoxicity, data are reported as geometric mean of fluorescence intensity. (**C**) Cells were incubated in the presence or absence (not induced, NI) of 0.3 μg/mL doxycycline for 2 days and analyzed for Tau expression by laser confocal microscopy (representative images on the left, scale bar 40 μm). Mean fluorescence intensity (MFI ± sem) in the presence of increasing amounts of doxycycline was determined by cytofluorimetry (graphs on the right, percent positive cells is shown).

To examine the subcellular distribution of Tau in primary human dermal fibroblasts, we fused in frame the cDNA of Tau_2N4R_ either with that of mCherry or of Gamillus (Fig. 1A), two differently emitting fluorescent proteins resistant to acidic pH^24^. A third cDNA construct was obtained by tagging Tau_2N4R_ with both mCherry and GFP (tandem-Tau) (Fig. 1A). This strategy takes advantage of the instability of GFP in an acidic environment exploited for monitoring LC3B subcellular distribution and autophagic flow^25^. The cDNAs were inserted into the pInducer20 vector for packaging into lentiviral pseudo particles^26^. Upon transduction for inducible expression of the single Tau variants, cells were characterized for Tau expression by confocal microscopy and flow cytometry. Protein expression was induced by cell treatment with doxycycline for 2 days. Doxycycline showed signs of cytotoxicity at concentrations above 2.5 μg/mL (Fig. 1B). Upon induction with 0.3 μg/mL doxycycline, all three fluorescent Tau variants were found in a pattern of cytosolic distribution consistent with the association of Tau to the microtubule network (Fig. 1C). Single cell analysis by flow cytometry showed a doxycycline dose-dependent increase in the amount of Tau present in the cell. Maximal induction levels were reached at about 0.3 μg/mL doxycycline. In the geneticin-resistant cell populations, the percentage of positive cells varied from 75% for tandem-Tau to 89% and 94% for Tau-mCherry and Tau-Gamillus, respectively (Fig. 1C). Only few cells expressed Tau in the absence of doxycycline indicating that the inducible system was not leaky. In the following experiments, Tau expression was routinely induced in the presence of 0.3 μg/mL doxycycline for 2 days.

### Tau localizes in DOs upon autophagy stimulation

**Figure 2:**
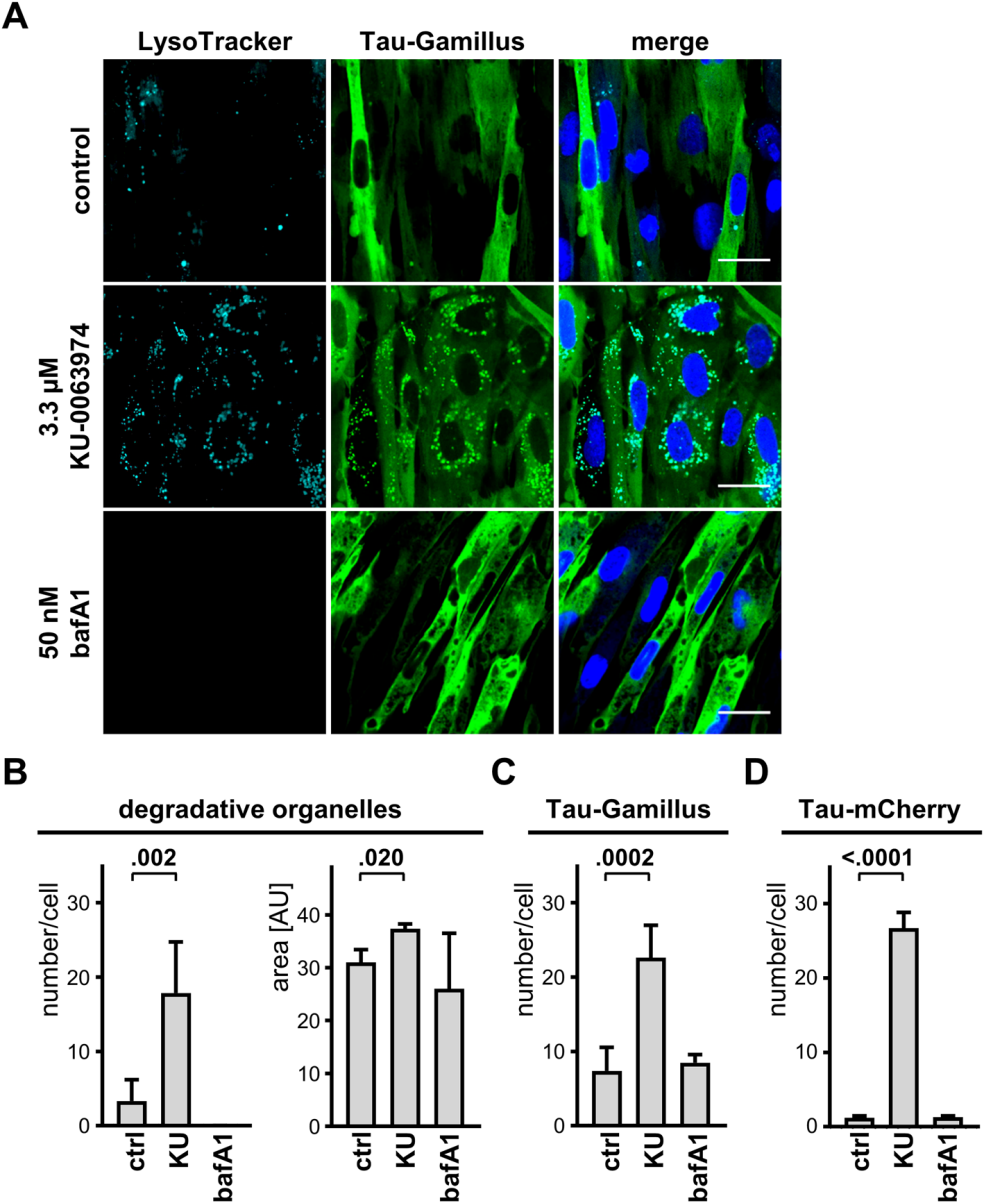
Tau is a target of the autophagic pathway. (**A**) Representative images by laser confocal microscopy of Tau-Gamillus fibroblasts cultured with 0.3 μg/mL doxycycline for 2 days. Cells were treated overnight with 3.3 μM of KU-0063974 (KU), 50 nM bafilomycin A1 (BafA1), or left untreated (control). Cells were stained with LysoTracker Red and Hoechst. Scale bar 47 μm. (**B**) Quantitative analysis of LysoTracker-positive DOs is shown in terms of DO number per cell (left graph, mean ± SD) and DO size (area, right graph, mean ± sem). (**C**) Quantitative analysis of Tau-Gamillus puncta number (mean ± SD) per cell expressing Tau-Gamillus. (**D**) Quantitative analysis of Tau-mCherry puncta number (mean ± SD) per cell expressing Tau-mCherry. Ordinary one-way ANOVA and Sidak’s multiple comparison test.

Pharmacologic manipulation of the ALP system was performed to assess whether Tau may represent a substrate of autophagy^27^ and be targeted to degradative organelles (DOs) of human fibroblasts. Autophagy was stimulated by mTOR inhibition with KU-0063794, a treatment expected to target most autophagy substrates to DOs^28^. In contrast, autophagy flux and DO acidification were inhibited with the vacuolar proton pump blocker bafilomycin A1^29^. Acidic DOs in Tau-Gamillus fibroblasts were labelled with the acidotrophic marker LysoTracker Red emitting red fluorescence. KU-0063794 treatment strongly increased the number of LysoTracker positive DOs, consistent with its action as stimulator of autophagy (Fig. 2A). Whereas under basal conditions Tau-Gamillus had a diffuse cytosolic pattern, the presence of KU-0063794 led to the appearance of Tau-Gamillus puncta that appeared colocalized with LysoTracker-positive DOs (Fig. 2A). In contrast, bafilomycin A1 eliminated the signal for the acidotrophic LysoTracker Red signal and did not induce the formation of Tau-Gamillus puncta (Fig. 2A). Quantitative laser confocal microscopy analysis proved that KU-0063794 increased the number and size of LysoTracker-positive DOs (Fig. 2B), as well as the mean number of Tau-Gamillus puncta per cell (Fig. 2C). Consistent results were obtained when analyzing Tau-mCherry fibroblasts with LysoTracker Green DND-26 (Fig. 2D). We concluded that Tau is a likely target of the autophagic pathway when ectopically expressed in primary human dermal fibroblasts. Although it is known that Tau is an autophagy substrate^30^, our experimental conditions cannot exclude the contribution of the biofluorescent markers fused to Tau for this observation.

### AD-derived brain seeds induce Tau accumulation in DOs

**Figure 3:**
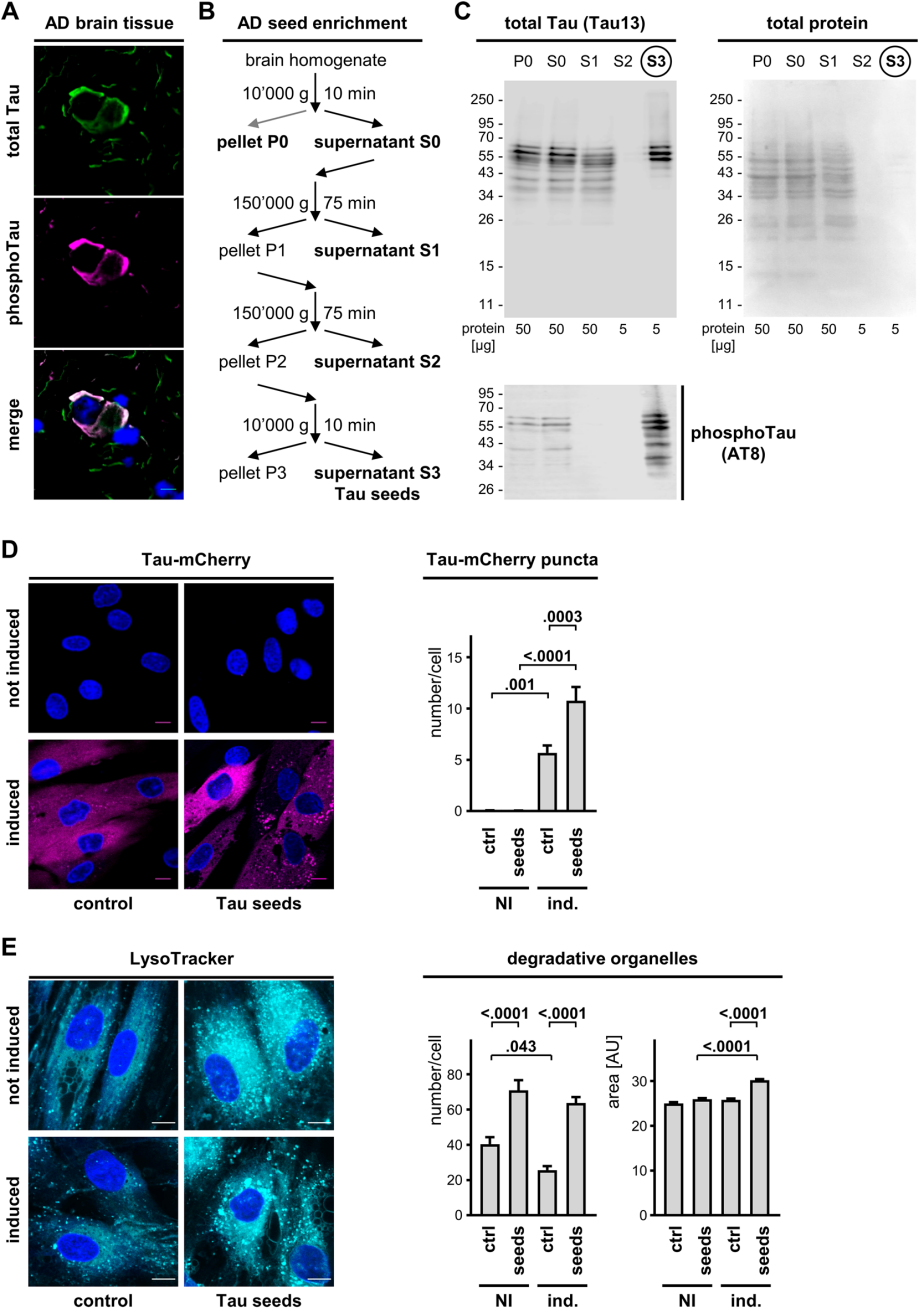
AD brain-derived Tau seeds induce Tau accumulation and lysosomal stress response. (**A**) Representative images by laser confocal microscopy of frozen AD brain sections stained for total Tau (revealed with anti-rabbit-AlexaFluor488 secondary antibody, in green) and for AT8 phosphoTau (revealed with anti-mouse-AlexaFluor594 secondary antibody, in magenta). Nuclear counterstaining with DAPI, scale bar 10 μm. (**B**) Scheme of the procedure used for enriching Tau seeds from frozen AD brain. (**C**) The indicated enrichment fractions were analyzed by western blot with the Tau13 antibody against total Tau or the AT8 antibody against phosphoTau. Primary antibodies were revealed with anti-mouse-IRDye800CW secondary antibody. Shown is also total protein staining with Ponceau S. (**D**) Representative images by laser confocal microscopy of Tau-mCherry fibroblasts. Cells were cultured with (induced) or without (not induced) 0.3 μg/mL doxycycline for a total of 4 days, whereby for the last 2 days cells were treated in the absence (control) or presence of AD brain-derived Tau seeds. Nuclei were counterstained with Hoechst, scale bar 30 μm. Shown is also the quantification of Tau-mCherry puncta number per cell (mean ± SD). (**E**) As in (**D**) but DOs were stained with LysoTracker. Shown is the DO number per cell (mean ± SD) and the DO size (mean ± sem). Ordinary one-way ANOVA and Sidak’s multiple comparison test.

We reported that fibrillogenic fragments of Tau carried by extracellular vesicles induced an aberrant accumulation of intracellular Tau within DOs of mouse neuronal C17.2 cells^27^. So, next we studied whether Tau accumulation may occur also in DOs of human primary fibroblasts incubated with fibrillogenic seeds. We isolated Tau seeds from postmortem human AD brain. The presence of Tau lesions in the AD brain tissue used for the fractionation was first verified by immune fluorescence staining with antibodies for total Tau. This evidenced robust Tau pathology in the form of neurofibrillary tangles and neuropil threads that were also positive for the triply phospho-epitope of Tau recognized by the AT8 antibody^31^ (Fig. 3A). An established differential centrifugation protocols^32,33^ was implemented to enrich Tau seeds starting from 2 g AD brain wet tissue (Fig. 3B). The initial P0 and S0 fractions accounted for ~ 120 mg and ~ 105 mg total protein, respectively, whereas the final S3 fraction contained ~ 1.7 mg total protein. The main fractions obtained were analyzed by western blot. The final supernatant S3 contained the largest relative amount of AT8-positive phosphorylated Tau when compared to the other fractions, although 10-time lower amounts of S3 protein were loaded on the gel (Fig. 3C), showing at least a 1000-fold enrichment of Tau seeds following this protocol.

Overnight addition of the Tau seeds to the culture medium of doxycycline-induced fibroblasts resulted in the robust accumulation of Tau-mCherry puncta in cells (Fig. 3D). Interestingly, also the number per cell of LysoTracker-positive DOs was increased (Fig. 3E). However, the effect of Tau seeds on DO number was not affected by the expression of Tau, indicating a lysosomal stress response caused by the treatment. In contrast, the increase of DO mean size required the presence of Tau (Fig. 3E), suggesting that intracellular Tau accumulation contributed to seed-induced lysosomal stress.

**Figure 4:**
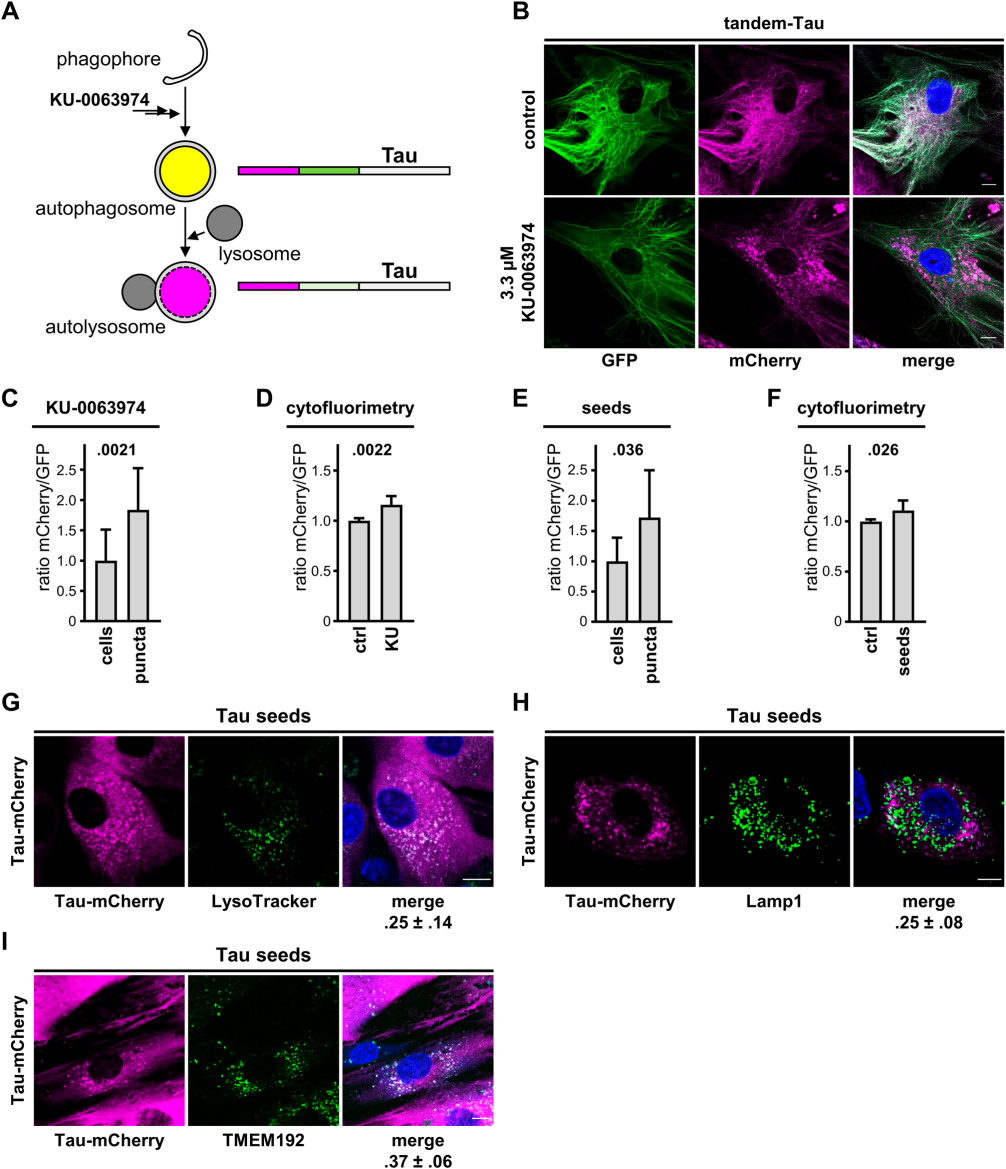
Tau accumulation occurs in DOs. (**A**) Principle of the tandem-Tau assay. (**B**) Representative images by laser confocal microscopy prior to fixation of Tandem-Tau fibroblasts induced with 0.3 μg/mL doxycycline for 2 days and then treated in the absence (control) or presence of 3.3 μM of the autophagy stimulator KU-0063974. Nuclei were counterstained with Hoechst, scale bar 20 μm. (**C**) Ratio of mCherry over GFP fluorescence (mean ± SD) calculated from confocal images utilizing a total cell mask (defined with an overlay of two emissions) or a tandem-Tau puncta mask (mCherry emission). (**D**) Ratio of mCherry over GFP (geometric mean fluorescence ± SD) determined by cytofluorimetry. (**E**,**F**) 2 days-induced tandem-Tau fibroblasts were treated overnight in the absence or presence of Tau seeds and analyzed as in (**C**,**D**). (**C**–**F**) Unpaired Mann Whitney *t*-test. (**G**,**H**) Representative laser confocal microscope images of mCherry-Tau fibroblasts (mCherry fluorescence in magenta) treated with Tau-seeds and stained (**G**) with LysoTracker (in green, nuclei counterstained with Hoechst in blue), or (**H**) with a LAMP1 antibody and a secondary anti-mouse AlexaFluor 488 antibody (in green, nuclei counterstained with DAPI in blue). (**I**) Representative images of doxycycline induced mCherry-Tau fibroblasts transduced for Gamillus-TMEM192 and incubated with Tau seeds overnight (TMEM192 in green, nuclei counterstained with Hoechst in blue). (**G**–**I**) Scale bar 30 μm. Under the merged images, the Pearson coefficient of colocalization is shown.

To further document Tau accumulation in DOs, we took advantage of the tandem-Tau system. Due to the instability of GFP in an acidic environment, we predicted that upon entry into acidic DOs, GFP emission would be lost but mCherry emission preserved (Fig. 4A), similarly to what described for the autophagy receptor LC3B^25^. Under control conditions, and upon induction of tandem-Tau expression with doxycycline, cells displayed GFP (in green) and mCherry (in magenta) fluorescence along the microtubule network (Fig. 4B). Treatment of the cells with the autophagy stimulator KU-0063794 again led to tandem-Tau accumulation in puncta that, yet, lacked GFP emission (Fig. 4B). Determination of the mCherry/GFP emission ratio for tandem-Tau puncta or for whole cells by quantitative laser confocal microscopy confirmed this property of tandem-Tau (Fig. 4C). Based on this observation, and to obtain more quantitative data, we next evaluated the use of cytofluorimetry as a mean to quantify the localization of Tau in acidic DOs. In the presence of KU-0063794, we observed a small, but statistically significant shift of the ratio of mean mCherry fluorescence over mean green fluorescence (Fig. 4D) by cytofluorimetry. The relatively small effect determined with this assay was possibly explained by double-fluorescent Tau present in the cytoplasm, which was partially masking the shift to mCherry fluorescence when Tau reached acidic DOs.

Prompted by the data obtained, we assessed the effect of Tau seeds when supplemented to tandem-Tau cells. We found that also in this case Tau seed-induced tandem-Tau puncta displayed increased mCherry/GFP emission ratio when analyzed by confocal microscopy (Fig. 4E) or by cytofluorimetry (Fig. 4F). These data indicated that Tau accumulation in fibroblasts occurred mainly in acidic DOs when induced by autophagy stimulation of treatment with extracellular Tau seeds. Consistent with this, accumulation of Tau co-localized with DOs positive for LysoTracker (Fig. 4G), for DOs stained with LAMP1 antibodies (Fig. 4H), and for DOs positive for the ectopic expression of TMEM192 (Fig. 4I). Overall, our data demonstrated aberrant accumulation of Tau in acidic DOs of primary human fibroblasts.

### Inhibition of GCase activity induces an increase of Tau accumulation in DOs

**Figure 5:**
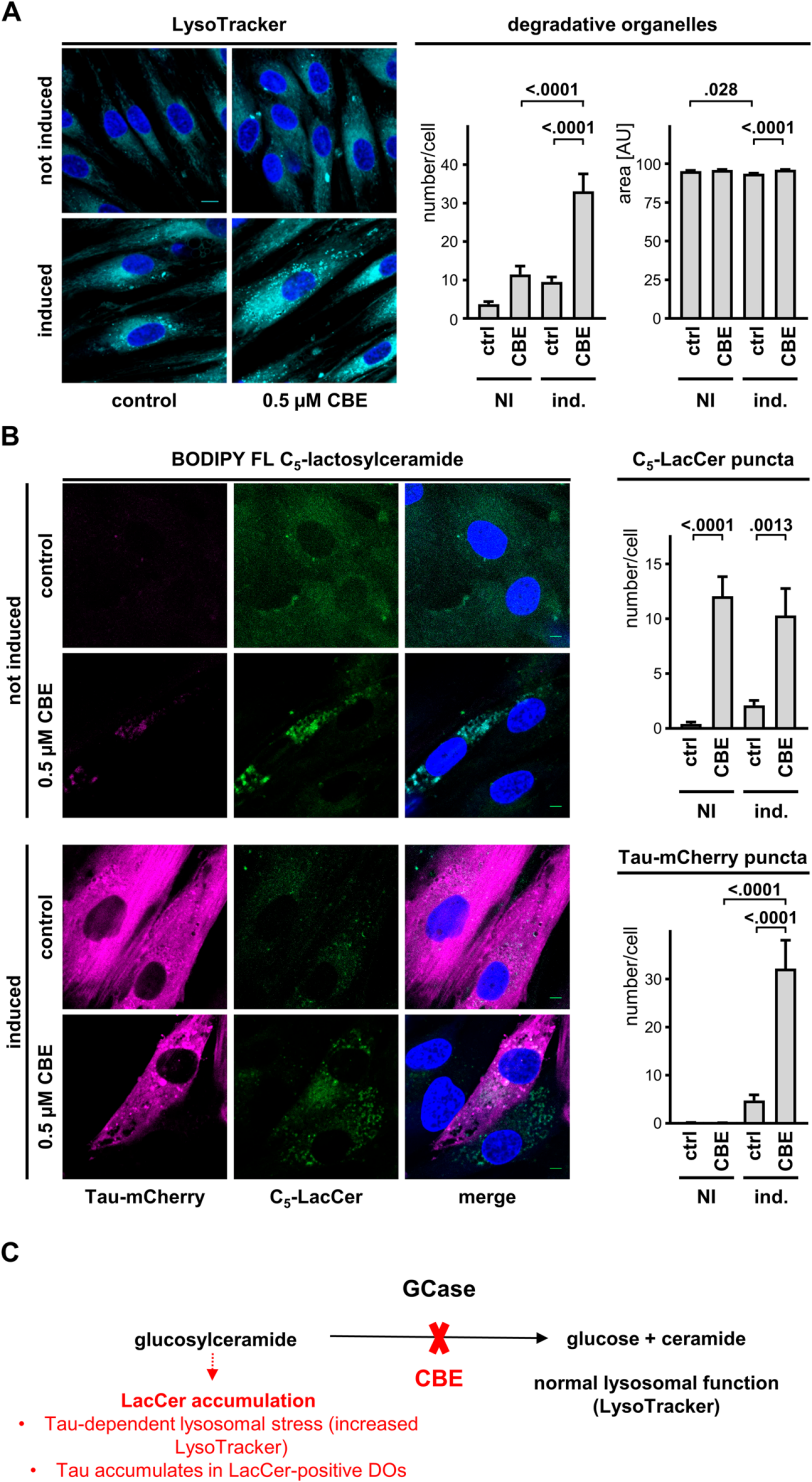
GCase inhibition promotes Tau accumulation and lysosomal stress. (**A**) Representative laser confocal microscope images (left) of 2 days-induced Tau-mCherry fibroblasts treated with 0.5 μM CBE overnight and stained with LysoTracker and Hoechst prior to fixation, scale bar 20 μm. Reported is the number per cells and size of LysoTracker-positive DOs (mean ± sem). Ordinary one-way ANOVA and Sidak’s multiple comparison test. (**B**) Tau-mCherry fibroblasts cultured in the absence (not induced) or presence (induced) of 0.3 μg/mL doxycycline were treated with 0.5 μM CBE overnight, incubated with 3.5 μM BODIPY FL C_5_-lactosylceramide for 15 min, and a day later analyzed by laser confocal microscopy. Representative images of Tau in magenta, C_5_-LacCer in green, and Hoechst in blue, scale bar 20 μm. Quantification of number per cell of C_5_-LacCer and Tau-mCherry puncta (mean ± sem). Ordinary one-way ANOVA and Sidak’s multiple comparison test. (**C**) Simplified schematic of the effect of GCase inhibition in the presence of CBE for the readout shown in the upper panels.

We rationalized that lysosomal stress, such as due to GCase impairment, may impact on the accumulation of Tau in DOs. So, we cultured the cells in the presence of conduritol-β-epoxide (CBE), an irreversible inhibitor of GCase. We first showed that one hour treatment with 0.5 µM CBE blocked GCase activity in fibroblasts. For this we used a cytofluorimetric assay for GCase activity with PFD-F dβGluP, a modified substrate of GCase that becomes fluorescent once metabolized by GCase (Suppl. Fig. 1). Overnight CBE treatment of fibroblasts expressing Tau-mCherry ultimately caused a cell response in terms of increased number of LysoTracker-positive DOs (Fig. 5A). In absence of Tau induction, we did not observe a statistically significant effect of CBE on DO number, thereby demonstrating a synergistic effect on the number of LysoTracker-positive DOs, a surrogate marker of lysosomal stress, due to the concomitant inhibition of GCase and the presence of Tau.

GCase impairment affects lipid metabolism in lysosomes^34,35^. Indeed, CBE treatment led to the appearance of DOs positive for BODIPY FL C_5_-lactosylceramide (Fig. 5B). The presence of Tau did not affect the CBE-mediated increase in BODIPY FL C_5_-lactosylceramide within DOs. However, Tau expression induced a CBE-dependent lysosomal stress, as judged by increased LysoTracker-positive DOs. Importantly, Tau-mCherry puncta accumulated in cells with CBE-inhibited GCase relative to non-inhibited controls (Fig. 5C), indicating that lysosomal dysfunction caused Tau accumulation.

### Seeded Tau accumulation in lysosomes induce lysosomal dysfunction/stress

**Figure 6:**
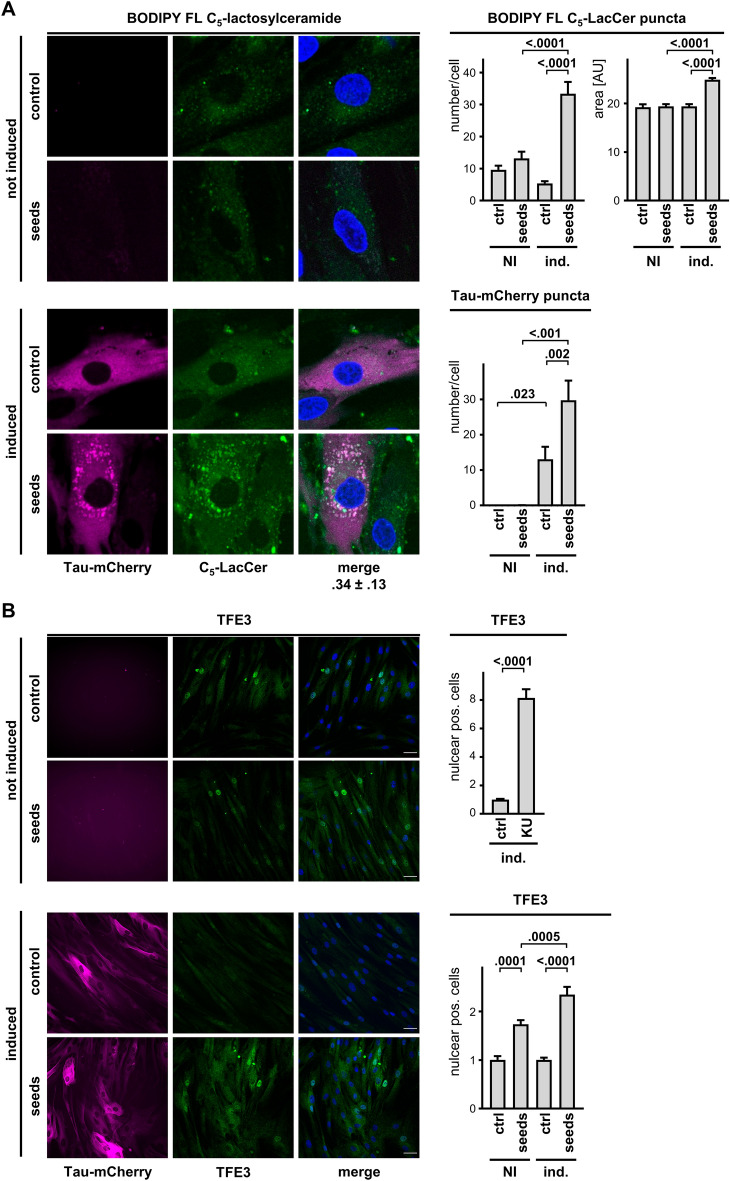
Seed-induced Tau accumulation is linked to lysosomal stress. Tau-mCherry fibroblasts cultured in the absence (not induced) or presence (induced) of 0.3 μg/mL doxycycline for 2 days were treated with Tau seeds overnight. (**A**) Representative images by laser confocal microscopy of cells (left) analyzed one day after incubation with 3.5 μM BODIPY FL C_5_-lactosylceramide for 15 min. Scale bar 20 μm. Shown under the merged image is the Pearson coefficient of colocalization. Quantification (right) of C_5_-LacCer puncta number per cell and size, and Tau-mCherry puncta number per cell (mean ± sem). Ordinary one-way ANOVA and Sidak’s multiple comparison test. (**B**) Representative images (left) of cells stained with a TFE3 antibody and a secondary anti-mouse AlexaFluor 488 antibody. Nuclei were counterstained with DAPI, scale bar 40 μm. Quantification (right) of percent cells with positive nuclear TFE3 after overnight incubation in the absence (ctrl) or presence of 3.3 μM KU-0063974 (upper graph, mean ± sem, unpaired Mann Whitney *t*-test), or Tau seeds (lower graph, mean ± sem, ordinary one-way ANOVA and Sidak’s multiple comparison test).

Next, we assessed the possible association of GCase inhibition to seed-induced Tau accumulation in DOs. First, we found that the addition of Tau seeds to Tau-mCherry expressing fibroblasts, but not in the absence of Tau expression induction, increased the formation of DOs positive for BODIPY FL C_5_-lactosylceramide. Again, Tau seeds promoted the accumulation of Tau-mCherry in puncta, which appeared to colocalize with the BODIPY FL C_5_-lactosylceramide positive DOs (Fig. 6A). These data indicated that Tau accumulation in the presence of extracellular AD brain seeds, induced a lysosomal dysfunction in terms of lipid metabolism as shown by the concomitant accumulation of BODIPY FL C_5_-lactosylceramide (Fig. 6A). A surrogate marker of lysosomal stress response is the nuclear translocation of TFE3, a master regulator potentiating lysosomal biogenesis and autophagic activity e.g., following treatment with the mTOR inhibitor KU-0063794 (Fig. 6B). Nuclear translocation of TFE3 was also observed when cells were incubated with Tau seeds, whereas Tau expression and Tau accumulation further increased this cell response. These data demonstrated that Tau accumulation in DOs contributed to a lysosomal stress response in primary fibroblasts.

## Discussion

We found evidence of aberrant Tau accumulation linked to lysosomal stress in primary human fibroblasts expressing various fluorescent forms of Tau. This adverse process was promoted by the presence of AD brain-derived Tau seeds or upon treatment with CBE: a cell-active pharmacologic irreversible inhibitor of lysosomal GCase. Tau was found to be a target for the autophagic pathway and Tau accumulation was colocalized with DO markers (LAMP1, TMEM192), the acidophilic compound LysoTracker, and showed a specific loss of GFP emission characteristic of an acidic environment. Based on our data, we propose that lysosomal dysfunction and the presence of internalized Tau seeds may cause, through an unknown mechanism, the accumulation of Tau on route to degradation within DOs. Similar results were described in murine cells incubated with exosomes containing a fibrillogenic fragment of Tau^27^. Tau accumulation in DOs was associated to increased lactosylceramide and lysosomal stress, indicating that seeded accumulation of Tau and impairment of lysosomal function and lipid metabolism are reciprocally engaged in a sequence of harmful events. Interestingly, Tau seed-mediated accumulation of lactosylceramide in DOs required Tau-expression. This may indicate a preferential patho-mechanism rendering more vulnerable neurons, which express high amounts of Tau.

CBE has been used to generate research models of GCase deficiency in LSD because the extent of GCase inactivation can be adjusted by variation in the inhibitor concentration and/or exposure time in cultured cells and mice^36^. Using a short overnight CBE treatment of human fibroblasts expressing Tau, we observed an accumulation of lactosylceramide and an increase of lysosome number and size. These data are in agreement with previous data reported in two neuronal models of GCase deficiency following a two-week CBE treatment that led to degeneration linked to increased glucosylceramide and glucosylsphingosine, nuclear translocation of TFE3, LAMP1 upregulation, enhanced lysosome size, number and exocytosis^37^. Also, SH-SY5Y cells treated with CBE presented abnormalities in autophagic flux, ALP, and mitochondrial activity^38,39^. We monitored Tau-dependent lipid accumulation and lysosomal dysfunction in primary fibroblasts and found that CBE-mediated lactosylceramide accumulation was independent on the presence of Tau. However, the increase of lysosome number and size, indicative of lysosomal stress, resulted from the concomitant accumulation of ectopically expressed Tau. Possibly because of the short treatment, the inhibition of GCase by CBE had a relatively weak effect on Tau accumulation, but showed, for the first time, a direct link between GCase dysfunction and Tau accumulation in acidic DOs. Accumulation of soluble and insoluble forms of α-synuclein following CBE treatment has been reported^38,40,41^ with some exceptions^42^.

The association between lysosomal impairment and accumulation of pathologic forms of Tau (aggregated and phosphorylated Tau) may contribute to pathology. Indeed, Tau lesions are present in a mouse model of Gaucher’s disease^43^, and other LSD such as Niemann–Pick^44^, Sanfilippo syndrome type B^45^, Christianson syndrome^46^ and Fabry’s disease^47^. Restoring mutated GCase activity with the chaperone Ambroxol^48^ or through ectopic expression of wild-type GCase^49^ delayed Tau and α-synuclein accumulation. These data support the importance of pharmacologic strategies aimed at increasing GCase activity for providing innovative, disease-modifying therapies for α-synucleinopathies and tauopathies. Promising are also genetic interventions correcting mutated GCase by SDM/CRISPR gene editing or overexpressing wild-type GCase^43^, as well as inhibitors of glucosylceramide synthase^50^.

ALP impairment as a consequence of protein aggregation may generate a vicious cycle boosting proteotoxicity in neurodegenerative disorders^9^. In addition, this may contribute to disease progression by favoring the transcellular spreading of pathogenic protein forms^51^ along neuro-anatomical connections by a prion-like mechanisms^52^. Indeed, injection of extracellular protein seeds in vivo induces intraneuronal pathology^53^ by a mechanism that hitherto remains a matter of debate^54^. Proposed are several mediators such as extracellular vesicles^55,56^, synaptic vesicle release^57^, membrane translocation^58^, clathrin-dependent endocytosis^59^, or transport of lysosome-associated aggregates through tunneling nanotubes^60,61^. A role of lysosomes in the propagation of pathogenic α-synuclein and Tau forms is supported by several studies^60,62–64^, emphasizing the need to better understand the link between (age-related) DOs dysfunction and protein aggregation. Our study supports a tight crosstalk between GCase activity, DOs dysfunction and aberrant Tau accumulation in DOs, offering new clues for treatment of tauopathies and LSD.

## Materials and methods

### Cell culture

Human primary dermal fibroblasts were isolated from a skin biopsy^65^ obtained from a healthy 30-year-old female. Fibroblasts were cultured in DMEM (61965-059, Gibco) supplemented with 15% FBS (F7524, BCBZ9153, Sigma), 1% non-essential amino acids (NEAA, 11140035, Gibco), 1% penicillin–streptomycin (P/S, 15140122, Gibco) and maintained at 37 °C in humidified atmosphere with 5% CO_2_. Cells were cultured for not more than 1 month. Human HEK293FT cells (gently provided by Prof. Barile, University of Southern Switzerland, Switzerland) were cultured in DMEM with 10% FBS (F7524, 0001643900 Sigma), 1% NEAA and 1% P/S.

### DNA plasmids

The plasmid pInducer20, a gift from Stephen Elledge (Addgene plasmid # 44012), was the vector for tet-on inducible expression of Tau_2N4R_ in human primary fibroblasts. The cDNAs (Supplementary Table 1) were amplified by the polymerase chain reaction (PCR) using human full-length templates and specific oligonucleotide primers from parental plasmids already available in the laboratory^27^. The cDNAs were first inserted in the pENTRY4 backbone (A10561, Invitrogen) before recombination with Gateway LR Clonase II (11791-020, Invitrogen) following the instructions of the manufacturer.

### Pseudoviral particle production and transduction

Pseudolentiviral particles were produced by transient transfection of HEK293FT cells with 2 μg of the desired pInducer plasmid and 8 μg packaging plasmid mix (pPACKH1-XL, LV510A-1, SBI). Cell conditioned medium was collected 2 days after transfection and cleared by centrifugation at 300*g* for 5 min, 4 °C. Pseudo-lentiviruses were 20-fold concentrated with centrifugal filters (MWCO 30 kDa, UFC903024, Amicon) at 3000*g* for 30–45 min, 4 °C, aliquoted and stored at − 80 °C.

Human primary fibroblasts (6–8 × 10^5^) were seeded into a 10 cm plate coated with poly-d-lysine (p6407, Sigma) one day before pseudolentiviral particle transduction. One day later, cells were supplemented with fresh complete medium and selected in the presence of 0.5 mg/mL geneticin (G418, 11811-031, Gibco) for 2 weeks.

### Drugs and cell treatments

Tau expression was induced with doxycycline (D9891, Sigma) for at least 2 days. Final drug concentrations were 3.3 μM for the mTORC1/mTORC2 complex inhibitor KU-0063794^28^ (HY-50710, Sigma), 20 nM for the lysosomal proton pump inhibitor bafilomycin A1 (BafA1, B1795, Sigma) and 0.5 μM for the lysosomal GCase inhibitor conduritol-beta-epoxide^66^ (CBE, 6090-95-5, Medchem).

### GCase activity

Human primary fibroblasts (3–5 × 10^5^) were seeded on poly-D-lysine coated MW6 or MW12, respectively. A day later cells were treated with 5-(pentafluorobenzoylamino) fluorescein di-β-d-glucopyranoside (PFB-FDGlu, P11947, Invitrogen) at 0.075 mM for 30 min, 37 °C. Cells were then gently washed with PBS and resuspended in 100–150 μL MACS buffer (PBS, 2% FBS, 2 mM EDTA) in a U bottom MW96. 100,000 cells were analyzed by cytofluorimetry.

### Cell and tissue histology

Cells expressing fluorescent Tau forms were seeded on poly-d-lysine coated 8 well microscope slides (80826-IBI, Ibidi). Nuclei were stained with 2.5 μg/mL Hoechst (H3570, Invitrogen) for 10 min, 37 °C, followed by gently washes in complete medium and PBS. Cells were fixed in 100% methanol for 20 min, − 20 °C and washed with PBS. Analysis and images were acquired on a microscope (ImageXpress 4 Micro, Molecular Devices). mCherry (ex: 587 nm, em: 610 nm) was visualized on the Texas Red channel (ex: 560/32 nm, em: 624/40 nm), GFP (ex: 488 nm, em: 507 nm) and Gamillus (ex: 504 nm, em 519) on the FITC channel (ex: 475/34 nm, em: 515/520 nm), and Hoechst (ex: 352 nm, em: 455 nm) with the DAPI channel (ex: 377/50, em: 461 nm).

For nuclear TFE3 analysis, Tau expression was first analyzed in live cells. Then, 4% formaldehyde fixed cells were stained with 0.3 μg/mL TFE3 antibody (HPA023881, Sigma), 2 μg/mL anti-rabbit IgG-Alexa488 antibody, nuclei were counterstained with DAPI. Analysis and images were acquired on the ImageXpress 4 Micro microscope. A DAPI nuclear mask was applied to determine the mean fluorescence intensity of nuclear TFE3 and outside the mask. Percentage of cells with a nuclear TFE3 phenotype was determined by applying an arbitrary threshold between the two masks. Total cell number was determined by counting the nuclei stained with DAPI for each image.

For LysoTracker staining, cells in poly-d-lysine 8 well slides were incubated with 0.25 μM LysoTracker (L7528 or L12492, ThermoFisher Scientific; 8783S, Cell Signaling) for 10 min, 37 °C followed by nuclear Hoechst counterstaining.

3.5 μM BODIPY FL C_5_-lactosylceramide complexed to BSA (B34402, Invitrogen) in ice-cold EBSS (24010-043, Gibco) was incubated on cells for 15 min, 4 °C. Cells were washed three times with ice-cold EBSS and then incubated in complete medium overnight, 37 °C. Before analysis, cells were fixed in 2% formaldehyde (F1635-4L, Sigma), and washed with 10 mM glycine in PBS.

For Lamp1 staining, cells were fixed in 4% formaldehyde for 5 min, 37 °C followed by 50% methanol for 10 min, room temperature. Cells were permeabilized and blocked with phosphate buffer, 10% normal goat serum, 15 mM glycine, 0.001% saponin (84510-100G, Sigma), 10 mM HEPES (15630-656, Gibco) for 1 h, room temperature. Cells were incubated in 0.2 μg/mL Lamp1 antibody (sc20011, Santa Cruz) for 1 h, 2 μg/mL anti-mouse IgG-Alexa488 antibody and DAPI.

Alzheimer’s disease frontal cortex samples were obtained from The Netherlands Brain Bank, Netherlands Institute for Neuroscience, Amsterdam (http://www.brainbank.nl). All anonymized donors signed a written informed consent for brain autopsy and further use of tissue and clinical information for research purpose. Based on local guidelines for research on anonymized samples, ethic committee authorization was not required. For immune staining, frozen tissue was cut in 5 μm sections with a cryostat (Cryostar NX50). Sections were fixed in 100% methanol (32213-5L, Sigma) for 15 min, − 20 °C. Fixed sections were incubated for 60 min in PBS with 5% normal goat serum (16210064, ThermoFisher Scientific) and 0.3% Triton X-100 (X100, Sigma-Aldrich). The sections were incubated overnight at 4 °C with 0.2 μg/mL TauAS rabbit antiserum or 0.4 μg/mL AT8 (MN1020, ThermoFisher Scientific) followed by 2 μg/mL secondary antibodies anti-rabbit IgG-Alexa488 (A-11034, ThermoFisher Scientific) or anti-mouse IgG-Alexa594 (A-11032) for 60 min, room temperature. Nuclei were counterstained with 0.5 μg/mL DAPI (D9542, Sigma-Aldrich) for 5 min, room temperature.

Images were acquired on a fluorescent laser confocal microscope (C2, Nikon) by sequential excitations (line-by-line scan) with the 405 nm laser (464/40 nm emission filter), the 488 nm laser (525/50 nm filter), and the 561 nm laser (561/LP nm filter).

### Enrichment of Tau seeds from AD brain tissue

Brain tissue pooled from 4–5 AD donors was homogenized on ice in ~ 9 volumes of filtered PHF buffer (10 mM Tris pH 7.4, 10% sucrose, 0.8 M NaCl and 0.1% sarkosyl, L9150-50G, Sigma) supplemented with protease and phosphatase inhibitor cocktails (S8820 and 04906845001, Sigma) in a glass Dounce homogenizer. Brain homogenates were briefly cleared by a centrifugation at 10,000*g* for 10 min, 4 °C. Sarkosyl was added to a 1% final concentration to the first supernatant (S0) and incubated for 90 min, room temperature, under agitation, before ultracentrifugation at 150,000*g* for 75 min, 10 °C. To remove sarkosyl, the P1 pellet was gently washed with cold PBS before repeating the ultracentrifugation. The P2 pellet was resuspended in 200 μL PBS supplemented with protease and phosphatase inhibitor cocktails and sonicated. The S2 supernatant was centrifuged at 10,000*g* for 10 min, 4 °C, and the Tau seed fraction S3 collected and stored frozen in aliquots. Total protein concentration was determined with the Pierce BCA protein assay kit (23227, ThermoFisher Scientific).

### Cell sorting and flow cytometry

For enrichment of double positive mCherry-GFP-Tau fibroblasts, 2 day-induced cells (3.5 × 10^6^) were first stained with 1/200 viability Aqua Dye (ex: 381 nm, em: 511 nm) (L34957, ThermoFisher Scientific) for 20 min, 37 °C. Cells were then washed, filtered through a 40 μm cell strainer (15-1040-2, Biologix) and resuspended in 1 mL cold MACS buffer before sorting (FACSymphony S6, BD Biosciences). Live cells were gated using DAPI channel (ex: 350 nm, em: 460 nm). Successively, selected live double positive cells were sorted for mCherry using the PECF594 channel (ex: 566 nm, em: 610 nm) and for GFP using the BB515 channel (ex: 490 nm, em: 515 nm).

For the GCase activity assay, cytofluorimetry (CytoFLEX, Beckman Coulter) was performed with the 488 nm excitation laser and 525/40 nm emission.

For the cytotoxicity assay, Aqua Dye-stained cells were analyzed by cytofluorimetry with 405 nm excitation laser and 450/45 nm emission.

For Tau expression, cytofluorimetry was acquired either with ex: 561 nm and em: 610/20 nm, or ex: 488 nm and em: 525/40 nm.

Data analysis was performed with the software FloJow (V 10.6.2, BD Biosciences). Values collected included total single cell number, gated cell number and geometric mean fluorescence.

### Western blot

Samples were diluted to 1 × SDS-PAGE sample buffer (1.5% SDS, 8.3% glycerol, 0.005% bromophenol blue, 1.6% β-mercaptoethanol, 62.5 mM Tris pH 6.8) and boiled for 10 min before 12% polyacrylamide gel electrophoresis. Resolved proteins were semi-dry transferred on a PVDF membrane, followed by incubation in 3% blocking buffer (Blocking Solution, 927-60001, Licor) in TBS for 1 h, room temperature. Primary antibodies were incubated overnight, 4 °C: 0.2 μg/mL Tau13-AlexaFluor (sc-21796 AF680, Santa Cruz) or 0.2 μg/mL AT8 (MN1020, ThermoFisher Scientific) and revealed with anti-mouse IgG IRDye RD 680 (Licor Biosciences, 926-68070) on an infrared imaging scanner (Licor Biosciences, Odyssey CLx 9140).

### Statistics and reproducibility

Statistical analysis was performed with GraphPad Prism version 8.4 with at least three independent biological replicates. Most quantifications are reported as fold over control/untreated conditions unless otherwise indicated in the graphs. Representative western blots and microscopic images are shown.

### Supplementary Information

Supplementary Information 1.

Supplementary Information 2.

Supplementary Figure 1.

Supplementary Table 1.

## Data Availability

All raw data can be found in the supplementary material.
