# Supplementary figures and images for: Tau accumulation in degradative organelles is associated to lysosomal stress
Source: Sci Rep. 2023 Oct 21;13:18024. doi: 10.1038/s41598-023-44979-7 (PMC10590387; doi:10.1038/s41598-023-44979-7)

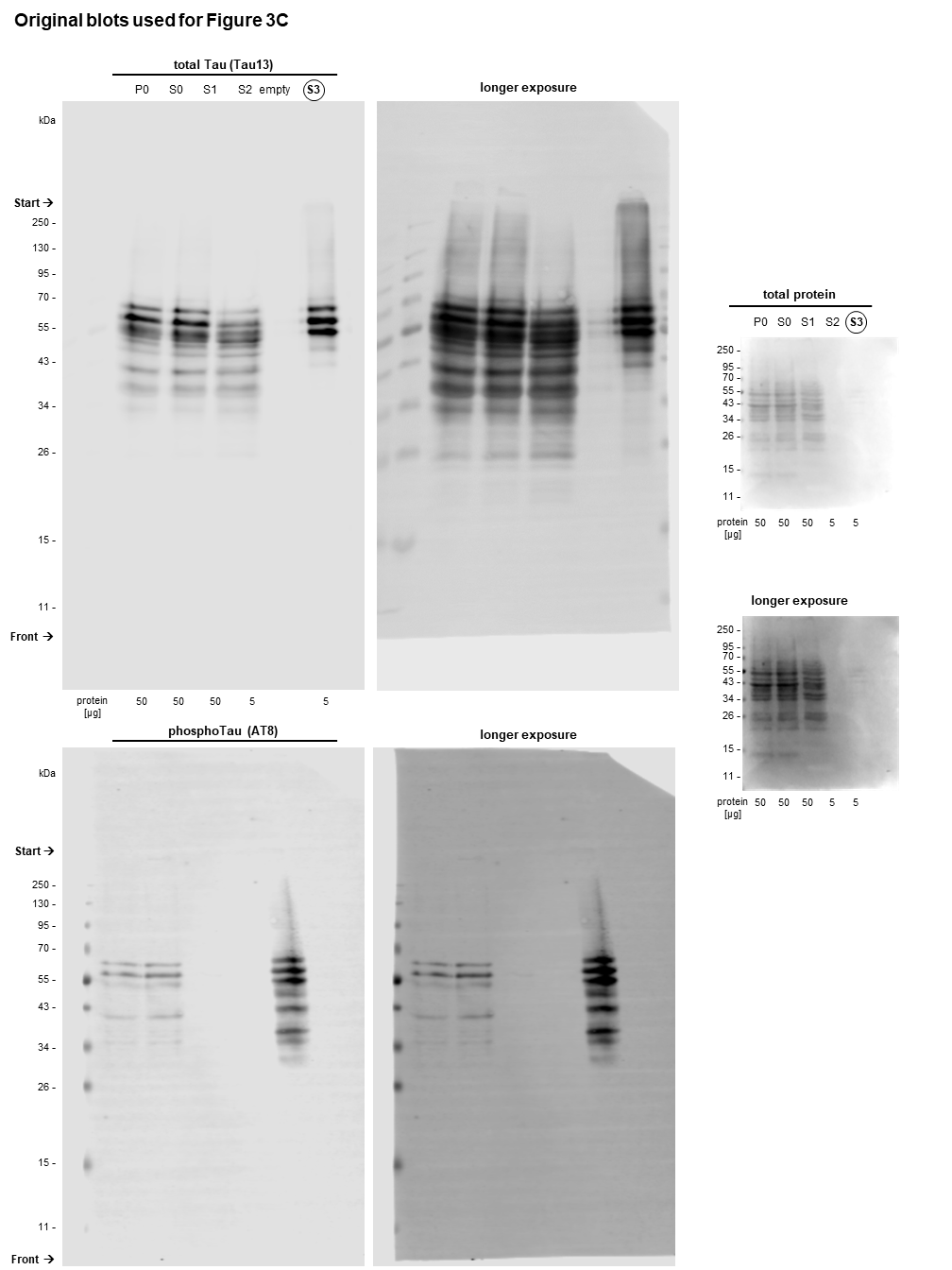

Supplement: Supplementary file 1 — Supplementary Information 1. [file 41598_2023_44979_MOESM1_ESM.tif]
