# Supplementary material for: Tau accumulation in degradative organelles is associated to lysosomal stress
Source: Sci Rep. 2023 Oct 21;13:18024. doi: 10.1038/s41598-023-44979-7 (PMC10590387; doi:10.1038/s41598-023-44979-7)
Supplement: Supplementary file 3 — Supplementary Figure 1. [file 41598_2023_44979_MOESM3_ESM.docx]

**Tau Accumulation in Degradative Organelles is Associated to Lysosomal Stress**

Ester Piovesana^1,2^, Claudia Magrin^1,2^, Matteo Ciccaldo^3^, Martina Sola^1,2^, Manolo Bellotto^4^, Maurizio Molinari^3,5^, Stéphanie Papin^1^, Paolo Paganetti^1, 2, 6, *^

^1^ Laboratory for Aging Disorders, Laboratories for Translational Research, Ente Ospedaliero Cantonale, Bellinzona, Switzerland

^2^ PhD Program in Neurosciences, Faculty of Biomedical Sciences, Università della Svizzera Italiana, Lugano, Switzerland

^3^ Institute for Research in Biomedicine, Faculty of Biomedical Sciences, Università della Svizzera italiana, Bellinzona, Switzerland

^4^ GT Gain Therapeutics SA, Lugano, Switzerland

^5^ School of Life Sciences, École Polytechnique Fédérale de Lausanne, Lausanne, Switzerland

^6^ Neurocentro della Svizzera Italiana, Ente Ospedaliero Cantonale, Lugano, Switzerland

* Corresponding author: Prof. Paolo Paganetti, Laboratories for Translational Research EOC, Room 102a, via Chiesa 5, CH-6500 Bellinzona, Switzerland

phone +41 58 666 7103

email: paolo.paganetti@eoc.ch or paolo.paganetti@usi.ch

***
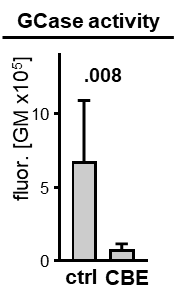
***

***Supplementary Figure 1.*** *Human primary fibroblasts were treated in the absence or presence of 1 mM GCase inhibitor CBE for 1.5 h. The GCase substrate PFB-FDGlu was added at 0.075 mM final concentration before analysis by cytofluorimetry for the determination of geometric mean fluorescence generated by the cleaved GCase substrate (± SD). Unpaired Mann Whitney t-test.*
