# Supplementary material for: Tau accumulation in degradative organelles is associated to lysosomal stress
Source: Sci Rep. 2023 Oct 21;13:18024. doi: 10.1038/s41598-023-44979-7 (PMC10590387; doi:10.1038/s41598-023-44979-7)
Supplement: Supplementary file 4 — Supplementary Table 1. [file 41598_2023_44979_MOESM4_ESM.docx]

**Tau Accumulation in Degradative Organelles is Associated to Lysosomal Stress**

Ester Piovesana^1,2^, Claudia Magrin^1,2^, Matteo Ciccaldo^3^, Martina Sola^1,2^, Manolo Bellotto^4^, Maurizio Molinari^3,5^, Stéphanie Papin^1^, Paolo Paganetti^1, 2, 6, *^

^1^ Laboratory for Aging Disorders, Laboratories for Translational Research, Ente Ospedaliero Cantonale, Bellinzona, Switzerland

^2^ PhD Program in Neurosciences, Faculty of Biomedical Sciences, Università della Svizzera Italiana, Lugano, Switzerland

^3^ Institute for Research in Biomedicine, Faculty of Biomedical Sciences, Università della Svizzera italiana, Bellinzona, Switzerland

^4^ GT Gain Therapeutics SA, Lugano, Switzerland

^5^ School of Life Sciences, École Polytechnique Fédérale de Lausanne, Lausanne, Switzerland

^6^ Neurocentro della Svizzera Italiana, Ente Ospedaliero Cantonale, Lugano, Switzerland

* Corresponding author: Prof. Paolo Paganetti, Laboratories for Translational Research EOC, Room 102a, via Chiesa 5, CH-6500 Bellinzona, Switzerland

phone +41 58 666 7103

email: paolo.paganetti@eoc.ch or paolo.paganetti@usi.ch

**Supplementary Table 1: cDNA sequences**

| Protein | cDNA sequence |
| --- | --- |
| *Tau-mCherry* | ATGGCTGAGCCCCGCCAGGAGTTCGAAGTGATGGAAGATCACGCTGGGACGTACGGGTTGGGGGACAGGAAAGATCAGGGGGGCTACACCATGCACCAAGACCAAGAGGGTGACACGGACGCTGGCCTGAAAGAATCTCCCCTGCAGACCCCCACTGAGGACGGATCTGAGGAACCGGGCTCTGAAACCTCTGATGCTAAGAGCACTCCAACAGCGGAAGATGTGACAGCACCCTTAGTGGATGAGGGAGCTCCCGGCAAGCAGGCTGCCGCGCAGCCCCACACGGAGATCCCAGAAGGAACCACAGCTGAAGAAGCAGGCATTGGAGACACCCCCAGCCTGGAAGACGAAGCTGCTGGTCACGTGACCCAAGCTCGCATGGTCAGTAAAAGCAAAGACGGGACTGGAAGCGATGACAAAAAAGCCAAGGGGGCTGATGGTAAAACGAAGATCGCCACACCGCGGGGAGCAGCCCCTCCAGGCCAGAAGGGCCAGGCCAACGCCACCAGGATTCCAGCAAAAACCCCGCCCGCTCCAAAGACACCACCCAGCTCTGGTGAACCTCCAAAATCAGGGGATCGCAGCGGCTACAGCAGCCCCGGCTCCCCAGGCACTCCCGGCAGCCGCTCCCGCACCCCGTCCCTTCCAACCCCACCCACCCGGGAGCCCAAGAAGGTGGCAGTGGTCCGTACTCCACCCAAGTCGCCGTCTTCCGCCAAGAGCCGCCTGCAGACAGCCCCCGTGCCCATGCCAGACCTGAAGAATGTCAAGTCCAAGATCGGCTCCACTGAGAACCTGAAGCACCAGCCGGGAGGCGGGAAGGTGCAGATAATTAATAAGAAGCTGGATCTTAGCAACGTCCAGTCCAAGTGTGGCTCAAAGGATAATATCAAACACGTCCCGGGAGGCGGCAGTGTGCAAATAGTCTACAAACCAGTTGACCTGAGCAAGGTGACCTCCAAGTGTGGCTCATTAGGCAACATCCATCATAAACCAGGAGGTGGCCAGGTGGAAGTAAAATCTGAGAAGCTTGACTTCAAGGACAGAGTCCAGTCGAAGATTGGGTCCCTGGACAATATCACCCACGTCCCTGGCGGAGGAAATAAAAAGATTGAAACCCACAAGCTGACCTTCCGCGAGAACGCCAAAGCCAAGACAGACCACGGGGCGGAGATCGTGTACAAGTCGCCAGTGGTGTCTGGGGACACGTCTCCACGGCATCTCAGCAATGTCTCCTCCACCGGCAGCATCGACATGGTAGACTCGCCCCAGCTCGCCACGCTAGCTGACGAGGTGTCTGCCTCCCTCGCGAAGCAGGGTTTGGTTTCGAAGGGCGAGGAGGATAACATGGCCATCATCAAGGAGTTCATGCGCTTCAAGGTGCACATGGAGGGCTCCGTGAACGGCCACGAGTTCGAGATCGAGGGCGAGGGCGAGGGCCGCCCCTACGAGGGCACCCAGACCGCCAAGCTGAAGGTGACCAAGGGTGGCCCCCTACCCTTCGCCTGGGACATCCTGTCCCCTCAGTTCATGTACGGCTCCAAGGCCTACGTGAAGCACCCCGCCGACATCCCCGACTACTTGAAGCTGTCCTTCCCCGAGGGCTTCAAGTGGGAGCGCGTGATGAACTTCGAGGACGGCGGCGTGGTGACCGTGACCCAGGACTCCTCCCTGCAGGACGGCGAGTTCATCTACAAGGTGAAGCTGCGCGGCACCAACTTCCCCTCCGACGGCCCCGTAATGCAGAAGAAGACCATGGGCTGGGAGGCCTCCTCCGAGCGGATGTACCCCGAGGACGGCGCCCTGAAGGGCGAGATCAAGCAGAGGCTGAAGCTGAAGGACGGCGGCCACTACGACGCTGAGGTCAAGACCACCTACAAGGCCAAGAAGCCCGTGCAGCTGCCCGGCGCCTACAACGTCAACATCAAGTTGGACATCACCTCCCACAACGAGGACTACACCATCGTGGAACAGTACGAACGCGCCGAGGGCCGCCACTCCACCGGCGGCATGGACGAGCTGTACAAGTAG |
| *Tau-Gamillus* | ATGGCTGAGCCCCGCCAGGAGTTCGAAGTGATGGAAGATCACGCTGGGACGTACGGGTTGGGGGACAGGAAAGATCAGGGGGGCTACACCATGCACCAAGACCAAGAGGGTGACACGGACGCTGGCCTGAAAGAATCTCCCCTGCAGACCCCCACTGAGGACGGATCTGAGGAACCGGGCTCTGAAACCTCTGATGCTAAGAGCACTCCAACAGCGGAAGATGTGACAGCACCCTTAGTGGATGAGGGAGCTCCCGGCAAGCAGGCTGCCGCGCAGCCCCACACGGAGATCCCAGAAGGAACCACAGCTGAAGAAGCAGGCATTGGAGACACCCCCAGCCTGGAAGACGAAGCTGCTGGTCACGTGACCCAAGCTCGCATGGTCAGTAAAAGCAAAGACGGGACTGGAAGCGATGACAAAAAAGCCAAGGGGGCTGATGGTAAAACGAAGATCGCCACACCGCGGGGAGCAGCCCCTCCAGGCCAGAAGGGCCAGGCCAACGCCACCAGGATTCCAGCAAAAACCCCGCCCGCTCCAAAGACACCACCCAGCTCTGGTGAACCTCCAAAATCAGGGGATCGCAGCGGCTACAGCAGCCCCGGCTCCCCAGGCACTCCCGGCAGCCGCTCCCGCACCCCGTCCCTTCCAACCCCACCCACCCGGGAGCCCAAGAAGGTGGCAGTGGTCCGTACTCCACCCAAGTCGCCGTCTTCCGCCAAGAGCCGCCTGCAGACAGCCCCCGTGCCCATGCCAGACCTGAAGAATGTCAAGTCCAAGATCGGCTCCACTGAGAACCTGAAGCACCAGCCGGGAGGCGGGAAGGTGCAGATAATTAATAAGAAGCTGGATCTTAGCAACGTCCAGTCCAAGTGTGGCTCAAAGGATAATATCAAACACGTCCCGGGAGGCGGCAGTGTGCAAATAGTCTACAAACCAGTTGACCTGAGCAAGGTGACCTCCAAGTGTGGCTCATTAGGCAACATCCATCATAAACCAGGAGGTGGCCAGGTGGAAGTAAAATCTGAGAAGCTTGACTTCAAGGACAGAGTCCAGTCGAAGATTGGGTCCCTGGACAATATCACCCACGTCCCTGGCGGAGGAAATAAAAAGATTGAAACCCACAAGCTGACCTTCCGCGAGAACGCCAAAGCCAAGACAGACCACGGGGCGGAGATCGTGTACAAGTCGCCAGTGGTGTCTGGGGACACGTCTCCACGGCATCTCAGCAATGTCTCCTCCACCGGCAGCATCGACATGGTAGACTCGCCCCAGCTCGCCACGCTAGCTGACGAGGTGTCTGCCTCCCTCGCGAAGCAGGGTTTGCTCGAGATGGTGAGCAAGGGCGAGGAGGCATCTGGCAGAGCCCTGTTCCAGTACCCCATGACCAGCAAGATCGAGCTGAACGGCGAGATCAACGGCAAGAAATTCAAGGTGGCCGGCGAGGGCTTCACCCCCAGCAGCGGCAGATTCAACATGCACGCCTACTGCACCACCGGCGACCTGCCTATGAGCTGGGTCGTGATTGCCAGCCCCCTCCAGTACGGCTTCCACATGTTCGCCCACTACCCCGAGGACATCACACACTTTTTCCAGGAATGCTTCCCCGGCAGCTACACCCTGGACCGGACCCTGAGAATGGAAGGCGACGGCACCCTGACCACCCACCACGAGTACAGCCTGGAGGACGGCTGCGTGACCTCCAAGACCACCCTGAATGCCAGCGGCTTCGACCCTAAGGGCGCCACCATGACCAAGAGCTTCGTGAAACAACTGCCTAACGAGGTGAAGATCACCCCCCACGGCCCCAACGGCATCAGACTGACCAGCACCGTGCTGTACCTGAAGGAGGATGGCACCATCCAGATCGGCACCCAGGACTGCATCGTGACCCCTGTGGGCGGAAGGAAAGTGACCCAGCCCAAGGCCCACTTCCTGCACACCCAGATCATCCAGAAGAAGGACCCCAACGACACCCGGGACCACATCGTGCAGACAGAACTGGCCGTGGCCGGCAATCTGTGGCACGGCATGGACGAGCTGTACAAG |
| *tandem-Tau* | ATGGCTGAGCCCCGCCAGGAGTTCGAAGTGATGGAAGATCACGCTGGGACGTACGGGTTGGGGGACAGGAAAGATCAGGGGGGCTACACCATGCACCAAGACCAAGAGGGTGACACGGACGCTGGCCTGAAAGAATCTCCCCTGCAGACCCCCACTGAGGACGGATCTGAGGAACCGGGCTCTGAAACCTCTGATGCTAAGAGCACTCCAACAGCGGAAGATGTGACAGCACCCTTAGTGGATGAGGGAGCTCCCGGCAAGCAGGCTGCCGCGCAGCCCCACACGGAGATCCCAGAAGGAACCACAGCTGAAGAAGCAGGCATTGGAGACACCCCCAGCCTGGAAGACGAAGCTGCTGGTCACGTGACCCAAGCTCGCATGGTCAGTAAAAGCAAAGACGGGACTGGAAGCGATGACAAAAAAGCCAAGGGGGCTGATGGTAAAACGAAGATCGCCACACCGCGGGGAGCAGCCCCTCCAGGCCAGAAGGGCCAGGCCAACGCCACCAGGATTCCAGCAAAAACCCCGCCCGCTCCAAAGACACCACCCAGCTCTGGTGAACCTCCAAAATCAGGGGATCGCAGCGGCTACAGCAGCCCCGGCTCCCCAGGCACTCCCGGCAGCCGCTCCCGCACCCCGTCCCTTCCAACCCCACCCACCCGGGAGCCCAAGAAGGTGGCAGTGGTCCGTACTCCACCCAAGTCGCCGTCTTCCGCCAAGAGCCGCCTGCAGACAGCCCCCGTGCCCATGCCAGACCTGAAGAATGTCAAGTCCAAGATCGGCTCCACTGAGAACCTGAAGCACCAGCCGGGAGGCGGGAAGGTGCAGATAATTAATAAGAAGCTGGATCTTAGCAACGTCCAGTCCAAGTGTGGCTCAAAGGATAATATCAAACACGTCCCGGGAGGCGGCAGTGTGCAAATAGTCTACAAACCAGTTGACCTGAGCAAGGTGACCTCCAAGTGTGGCTCATTAGGCAACATCCATCATAAACCAGGAGGTGGCCAGGTGGAAGTAAAATCTGAGAAGCTTGACTTCAAGGACAGAGTCCAGTCGAAGATTGGGTCCCTGGACAATATCACCCACGTCCCTGGCGGAGGAAATAAAAAGATTGAAACCCACAAGCTGACCTTCCGCGAGAACGCCAAAGCCAAGACAGACCACGGGGCGGAGATCGTGTACAAGTCGCCAGTGGTGTCTGGGGACACGTCTCCACGGCATCTCAGCAATGTCTCCTCCACCGGCAGCATCGACATGGTAGACTCGCCCCAGCTCGCCACGCTAGCTGACGAGGTGTCTGCCTCCCTCGCGAAGCAGGGTTTGCTCGAGATGGTGAGCAAGGGCGAGGAGGCATCTGGCAGAGCCCTGTTCCAGTACCCCATGACCAGCAAGATCGAGCTGAACGGCGAGATCAACGGCAAGAAATTCAAGGTGGCCGGCGAGGGCTTCACCCCCAGCAGCGGCAGATTCAACATGCACGCCTACTGCACCACCGGCGACCTGCCTATGAGCTGGGTCGTGATTGCCAGCCCCCTCCAGTACGGCTTCCACATGTTCGCCCACTACCCCGAGGACATCACACACTTTTTCCAGGAATGCTTCCCCGGCAGCTACACCCTGGACCGGACCCTGAGAATGGAAGGCGACGGCACCCTGACCACCCACCACGAGTACAGCCTGGAGGACGGCTGCGTGACCTCCAAGACCACCCTGAATGCCAGCGGCTTCGACCCTAAGGGCGCCACCATGACCAAGAGCTTCGTGAAACAACTGCCTAACGAGGTGAAGATCACCCCCCACGGCCCCAACGGCATCAGACTGACCAGCACCGTGCTGTACCTGAAGGAGGATGGCACCATCCAGATCGGCACCCAGGACTGCATCGTGACCCCTGTGGGCGGAAGGAAAGTGACCCAGCCCAAGGCCCACTTCCTGCACACCCAGATCATCCAGAAGAAGGACCCCAACGACACCCGGGACCACATCGTGCAGACAGAACTGGCCGTGGCCGGCAATCTGTGGCACGGCATGGACGAGCTGTACAAG |
| *Gamillus-TMEM192-3HA* | ATGGCTGAGCCCCGCCAGGAGTTCGAAGTGATGGAAGATCACGCTGGGACGTACGGGTTGGGGGACAGGAAAGATCAGGGGGGCTACACCATGCACCAAGACCAAGAGGGTGACACGGACGCTGGCCTGAAAGAATCTCCCCTGCAGACCCCCACTGAGGACGGATCTGAGGAACCGGGCTCTGAAACCTCTGATGCTAAGAGCACTCCAACAGCGGAAGATGTGACAGCACCCTTAGTGGATGAGGGAGCTCCCGGCAAGCAGGCTGCCGCGCAGCCCCACACGGAGATCCCAGAAGGAACCACAGCTGAAGAAGCAGGCATTGGAGACACCCCCAGCCTGGAAGACGAAGCTGCTGGTCACGTGACCCAAGCTCGCATGGTCAGTAAAAGCAAAGACGGGACTGGAAGCGATGACAAAAAAGCCAAGGGGGCTGATGGTAAAACGAAGATCGCCACACCGCGGGGAGCAGCCCCTCCAGGCCAGAAGGGCCAGGCCAACGCCACCAGGATTCCAGCAAAAACCCCGCCCGCTCCAAAGACACCACCCAGCTCTGGTGAACCTCCAAAATCAGGGGATCGCAGCGGCTACAGCAGCCCCGGCTCCCCAGGCACTCCCGGCAGCCGCTCCCGCACCCCGTCCCTTCCAACCCCACCCACCCGGGAGCCCAAGAAGGTGGCAGTGGTCCGTACTCCACCCAAGTCGCCGTCTTCCGCCAAGAGCCGCCTGCAGACAGCCCCCGTGCCCATGCCAGACCTGAAGAATGTCAAGTCCAAGATCGGCTCCACTGAGAACCTGAAGCACCAGCCGGGAGGCGGGAAGGTGCAGATAATTAATAAGAAGCTGGATCTTAGCAACGTCCAGTCCAAGTGTGGCTCAAAGGATAATATCAAACACGTCCCGGGAGGCGGCAGTGTGCAAATAGTCTACAAACCAGTTGACCTGAGCAAGGTGACCTCCAAGTGTGGCTCATTAGGCAACATCCATCATAAACCAGGAGGTGGCCAGGTGGAAGTAAAATCTGAGAAGCTTGACTTCAAGGACAGAGTCCAGTCGAAGATTGGGTCCCTGGACAATATCACCCACGTCCCTGGCGGAGGAAATAAAAAGATTGAAACCCACAAGCTGACCTTCCGCGAGAACGCCAAAGCCAAGACAGACCACGGGGCGGAGATCGTGTACAAGTCGCCAGTGGTGTCTGGGGACACGTCTCCACGGCATCTCAGCAATGTCTCCTCCACCGGCAGCATCGACATGGTAGACTCGCCCCAGCTCGCCACGCTAGCTGACGAGGTGTCTGCCTCCCTCGCGAAGCAGGGTTTGCTCGAGATGGTGAGCAAGGGCGAGGAGGCATCTGGCAGAGCCCTGTTCCAGTACCCCATGACCAGCAAGATCGAGCTGAACGGCGAGATCAACGGCAAGAAATTCAAGGTGGCCGGCGAGGGCTTCACCCCCAGCAGCGGCAGATTCAACATGCACGCCTACTGCACCACCGGCGACCTGCCTATGAGCTGGGTCGTGATTGCCAGCCCCCTCCAGTACGGCTTCCACATGTTCGCCCACTACCCCGAGGACATCACACACTTTTTCCAGGAATGCTTCCCCGGCAGCTACACCCTGGACCGGACCCTGAGAATGGAAGGCGACGGCACCCTGACCACCCACCACGAGTACAGCCTGGAGGACGGCTGCGTGACCTCCAAGACCACCCTGAATGCCAGCGGCTTCGACCCTAAGGGCGCCACCATGACCAAGAGCTTCGTGAAACAACTGCCTAACGAGGTGAAGATCACCCCCCACGGCCCCAACGGCATCAGACTGACCAGCACCGTGCTGTACCTGAAGGAGGATGGCACCATCCAGATCGGCACCCAGGACTGCATCGTGACCCCTGTGGGCGGAAGGAAAGTGACCCAGCCCAAGGCCCACTTCCTGCACACCCAGATCATCCAGAAGAAGGACCCCAACGACACCCGGGACCACATCGTGCAGACAGAACTGGCCGTGGCCGGCAATCTGTGGCACGGCATGGACGAGCTGTACAAG |
